# Mitral valve repair versus mitral valve replacement in octogenarians - review of long-term outcomes in the past two decades

**DOI:** 10.1186/1749-8090-10-S1-A341

**Published:** 2015-12-16

**Authors:** Miriam Silaschi, Sanjay Chaubey, Habib Khan, Mohsin Uzzaman, Mrinal Singh, Max Baghai, Olaf Wendler

**Affiliations:** 1 Department of Cardiothoracic Surgery, King's College Hospital London, London, UK

## Background/Introduction

During the past decades, the cardiovascular community was faced with an ageing patient population and thus with an increased number of elderly patients referred for cardiac surgery. This is also perceptible for mitral valve (MV) disease, as gold standard treatment is MV-repair or replacement. Satisfactory results were proven in octogenarians, but comparative data of both treatments is scarce.

## Aims/Objectives

We reviewed results after either MV-repair or replacement in octogenarians treated at our centre over the past 20 years.

## Method

Our in-hospital database was explored for patients who had MV surgery; this yielded 1736 patients treated between 1994 and 2014. 155 patients (8.9%) were aged ≤80 years and received MV-repair (n = 106,68.4%) or replacement (MVR: n = 49,31.6%). In 53.8% of MV-repair and 51.0% of MVR concomitant procedures were performed. A comparative survival-analysis of octogenarians with adjustment for valve pathology and a subgroup-analysis for isolated procedures was performed.

## Results

Mean age was 82.1 ± 1.9yrs (MV-repair) vs 82.5 ± 2.3yrs (MVR: p = 0.21). Logistic EuroSCORE was 19.7 ± 6.6% vs. 20.9 ± 14.9% (p = 0.73). Median follow-up was 918.5 days (IQR 272.5-1862). Thirty-day mortality was 7.5% (MV-repair) vs. 12.2% (MVR: p = 0.37). ICU-stay was 46.8 ± 118.8 hrs (MV-repair) vs. 48.8 ± 43.9 hrs (MVR: p = 0.89); ventilation time was (Median [IQR]) 6 [5-11.5] vs. 9 [6-14.5]hrs (p = 0.83). Adjusted 1-,2-,5- and 8 year survival was 88.3%, 83.2%, 66.6% and 32.2% after MV-repair vs. 68.6%, 63.2%, 39.6% and 19.0% (MVR; p = 0.02). 1-,2-,5- and 8 year survival of isolated procedures was 91.7%, 84.9%, 74.3% and 35.2% after MV-repair vs. 78.3%, 67.4%, 34.1% and 18.2% after MVR (p = 0.03).

## Discussion/Conclusion

Long-term survival after MV-repair was superior to MVR. This was still apparent after adjustment for valve pathology and in the subgroup of isolated procedures. Even in octogenarians, the complex anatomy of the mitral valve should be preserved and the valve repaired whenever possible.

